# Recruitment and engagement of a cohort of women living with HIV in Nigeria: Baseline characteristics from the Nigeria Implementation Science Alliance

**DOI:** 10.1371/journal.pgph.0004028

**Published:** 2025-04-16

**Authors:** Nwamaka Alexandra Ezeonu, John Olajide Olawepo, Uche Okezie, Emmanuel Egbo, Ijeoma Uchenna Itanyi, Ahmad Aliyu, Tonia C. Onyeka, Babayemi Oluwaseun Olakunde, Collins Imarhiagbe, Stephen Tersoo Orafa, Samuel Cheure, Uduak Akpan, Echezona Edozie Ezeanolue

**Affiliations:** 1 IVAN Research Institute, University of Nigeria, Enugu, Nigeria; 2 Department of Public Health and Health Sciences, Northeastern University, Boston, Massachusetts, United States of America; 3 APIN Public Health Initiatives, Abuja, FCT, Nigeria; 4 Department of Community Medicine, University of Nigeria, Enugu, Nigeria; 5 Dalla Lana School of Public Health, University of Toronto, Toronto, Canada; 6 Institute of Human Virology Nigeria, Abuja, FCT, Nigeria; 7 Department of Anaesthesia, Pain and Palliative Care Unit, Faculty of Clinical Sciences, University of Nigeria, Enugu, Nigeria; 8 Department of Population and Community Health, School of Public Health, University of North Texas Health Science Center, Fort Worth, Texas, United States of America,; 9 Centre for Integrated Health Programs (CIHP), Abuja, FCT, Nigeria; 10 Caritas Nigeria, Abuja, FCT, Nigeria; 11 Excellence Community Education Welfare Scheme (ECEWS), Akwa Ibom, Nigeria; 12 Healthy Sunrise Foundation, Las Vegas, Nevada, United States of America; Duke University, UNITED STATES OF AMERICA

## Abstract

Nigeria has a high burden of mother to child transmission (MTCT) of HIV. There is paucity of large-scale prospective cohort studies to provide insight into the reasons for the abysmal MTCT indices. This paper describes the baseline characteristics of women living with HIV who signed consent to participate in future clinical or implementation trials. The Nigeria Implementation Science Alliance (NISA) developed an open multicentre prospective cohort of women of reproductive age living with HIV, drawn from 12 facilities across the six geo-political regions of Nigeria. Research Electronic Data Capture system was used for the informed consent process. Socio-demographic and clinical information of participants were accessed through the clinics’ Electronic Medical Records. We calculated descriptive statistics, summarizing categorical variables using frequencies and percentages. Numerical variables were summarized using means and standard deviations for normally distributed, and median and interquartile ranges for skewed variables. We recruited 18,210 women living with HIV. Eighty-one percent (14,777/18,210) had their data extracted from the EMR. Data of 10,996 women were analysed. The mean age was 37.4 ± 7.2 years, with 85% in age groups ≥30–39 years. The median time since HIV diagnosis was 8 years (IQR 3–11 years) while the median length of time on ART was 6 years (IQR 3–10 years). For women who had a record of WHO clinical staging and most current viral load, majority (80%) were in WHO stage 1 while two thirds (68.0%) had viral load of <20 copies/mm^3^. Almost all women (94%) were on first-line antiretrovirals, with none on the third-line regimen. This unique cohort in Nigeria that will provide researchers with a platform to propose and answer several research questions about the health of women and infants providing policymakers with information on maternal and child health in Nigeria.

## Introduction

Nigeria has made steady progress towards the UNAIDS 95-95-95 goals having diagnosed 90% of persons living with HIV, placed 90% on anti-retroviral therapy (ART) with 86% achieving viral suppression [1]. Nevertheless, there is poor progress towards the Elimination of Mother to Child Transmission (EMTCT) of HIV [2]. The World Health Organization (WHO) minimum impact target for the EMTCT of HIV is a transmission rate of < 5% among the breastfed and < 50 paediatric infections per 100,000 live births [3]. Nigeria presently lags behind with a final MTCT rate of 25%, 15% in the first six weeks of life, and 10% during breastfeeding [4].

Based on the latest demographic and health survey, only 67% of pregnant women in Nigeria had at least one antenatal visit [5]. With an estimated 40 million women of childbearing age [6], 8 million annual pregnancies [7], and 7 million babies delivered annually in Nigeria [6], only 33% (2.3 million) of the mothers get tested for HIV at the antenatal clinics [7]. Of the women living with HIV who enter PMTCT cascade of care, about two-thirds are retained in care [8], although a range of 41% to 88% has been reported in other studies [9–12]. In 2021, 34% of HIV-infected pregnant women accessed antiretroviral drugs for PMTCT down from 53% in 2015 and far below the UNAIDS target of 95% [1,13].

Despite the 2020 global target of 20,000 new paediatric infections, Nigeria had an estimated 26,000 new paediatric HIV infections in 2021, the highest in the world, and is responsible for 14% of the global burden of MTCT of HIV [1,14–16]. Although the number of new annual HIV infections among children in Nigeria was reduced by half between 2014 and 2020[1,17], the incidence of annual new paediatric infection is projected to increase to 44,823 by 2030[18], and significant gaps remain along the PMTCT continuum of care to achieve the elimination targets [19,20].

Early Infant Diagnosis (EID) is essential for improving interventions for exposed infants. The proportion of Nigerian children tested for HIV at two months of age is low (15%), and much lower when breastfeeding ends because they are lost to follow-up [1,17]. In 2020, only 31% of the 170,000 children living with HIV in Nigeria were receiving ART, with 17,000 deaths among the children living with HIV aged 0–14 years [1,21]. Retention of mother-infant pairs along the PMTCT continuum of care remains a major problem in Nigeria, largely due to just over a third of deliveries in Nigeria occurring in formal health facility settings [5,22]. The disruption of health services occasioned by the COVID-19 pandemic may yet worsen these gaps [23,24].

Despite the huge MTCT burden, the paucity of large-scale prospective cohort studies to provide insight into the reasons for the abysmal MTCT indices is of public health concern. In spite of the significant investments, gaps in local research infrastructure [25,26] have limited the availability of prospective longitudinal studies to better understand the epidemiology and risk factors, and inform interventions towards achieving the goal of EMTCT in Nigeria.

To contribute towards addressing these problems, the Nigeria Implementation Science Alliance (NISA) set out two main goals: First, it identified 21 comprehensive HIV treatment health facilities across the six regions of the country to serve as Implementation Laboratories named Model Innovation and Research Centers (MIRCs) [27]. Second, it set out to recruit, engage, and retain a large, diverse cohort of consented participants across 12 of the 21 NISA-MIRCs, with two centers selected from each of the country’s six regions. Research activities at the NISA-MIRCs are coordinated by the IVAN Research Institute at the College of Medicine, University of Nigeria.

This paper describes the baseline characteristics of women from the 12 sites who signed consent to participate in the NISA Women and Infant Cohort (NISA-WIC) and consented to be informed when a clinical or implementation study is available for them to participate. Researchers and collaborators can leverage this cohort to propose and answer relevant research questions by collecting relevant data on participants after ethical approvals.

## Methods

### Ethics statement

This study was approved by the National Health Research Ethics Committee of Nigeria - NHREC/01/01/2007-25/03/2021. The adult participants gave verbal informed consent. Participants below 18 years gave assent, and their parent or guardian provided verbal informed consent on their behalf. Research staff and health facility staff participated in a training on ‘Informed Consent and Research Ethics’ before study commencement to ensure the highest ethical standards.

### Study area

Nigeria consists of thirty-six states and the Federal Capital Territory, divided into six geopolitical regions (South-East, South-South, South-West, North-Central, North-East, and North-West,) The country is very diverse with over 250 ethnic groups who speak over 500 indigenous languages [28,29]. Nigeria’s population is estimated to be about 219 million, making it the sixth most populous nation globally in 2021 [29]. HIV prevalence rate among 15–49 years old is 1.3% [1].

### Study design and study population

The NISA Women and Infant Cohort (NISA-WIC) is an open multicentre prospective cohort of women of reproductive age living with HIV. The cohort is drawn from 12 selected HIV treatment health facilities supported by the US President’s Emergency Plan for AIDS Relief (PEPFAR) across the six geo-political regions of Nigeria. Five leading local implementing partners support these 12 sites, namely APIN Public Health Initiatives Nigeria (APIN), Institute of Human Virology, Nigeria (IHVN), Centre for Integrated Health Programmes (CIHP), Caritas Nigeria (CCFN), and Excellence Community Education Welfare Scheme (ECEWS). The details for the selection of the HIV treatment facilities have been described in a related previous publication [27].

### Eligibility criteria

The criteria for recruitment of participants into the cohort included women: (a) aged between 15–49 years; (b) receiving ART for at least six months at the participating health facilities; (c) who had a functional contact phone number; and (d) who gave their informed consent to join the cohort.

### Recruitment procedure

The cohort of women living with HIV was built from the pooled list of all women of child-bearing age who receive HIV treatment at the 12 selected sites. From 31/08/2021 to 23/05/2022, during routine clinic visits, the health facility staff identified clients who met the eligibility criteria. The clinic staff (nurse or doctor) informed the eligible woman living with HIV about the cohort and, if interested, directed them to the data clerk who explained details of the cohort study and answered any questions. Verbal informed consent was provided by the adult participants. For women 15–17 years, verbal assent was provided by participants while a parent/guardian provided verbal informed consent. Consent information was presented in either English or the local language, as necessary. Data obtained during the consent process included hospital name, participants’ hospital number, participants’ name and initial, consent officers’ name and initials and date. The Research Electronic Data capture system (REDCap) installed on a computer tablet was used for the informed consent process. The database was hosted by a certified cloud service provider with a signed Business Associate Agreement (BAA) covering the Health Insurance Portability and Accountability Act (HIPAA). A WhatsApp^®^ group was created for communication and updates [30].

### Data sources, management and security plan

Following enrollment into the cohort, the consent data was de-duplicated, then the participants’ socio-demographic and clinical information were accessed through the clinics’ electronic medical records (EMR) on 24/05/2022. The Strategic Information leads of the implementing partners (IPs) led the extraction process from the EMR for their supported facilities. They supervised the coding process and verified the data that were extracted.

The data from the 12 facilities were merged on Excel by the NISA Information Technology lead and validated by 2 of the authors. The extracted EMR data was cleaned and cross-checked for accuracy and errors then de-identified. The data was exported for analysis with International Business Machines Statistical Package for the Social Sciences (IBM SPSS) software version 25. All the data were password-protected and stored on a password-protected computer. Access to the data was limited to those permitted by the Principal Investigator. Appropriate measures were taken to protect data in transit and at rest.

### Data analyses

We calculated descriptive statistics, summarizing categorical variables using frequencies and proportions. Continuous variables were summarized using means and standard deviations for normally distributed, and median and interquartile ranges for skewed variables. Age was analysed as a continuous variable using mean and standard deviation and recategorized using age groups. Marital status, level of completed education, parity, pregnancy status, WHO clinical staging, Body Mass Index (BMI) [weight/height^2^], viral load, and line of regimen were analysed as categorical variables. Weight, height, length of time in years since HIV diagnosis, and length of time in years since the start of ART were analysed as continuous variables. For each variable, the missing values were reported in the results. Littles’ MCAR was significant (p<0.001) which showed that the data were not missing completely at random (p<0.001). We did not conduct further analysis as this manuscript is a description of the cohort.

## Results

**Fig 1:**
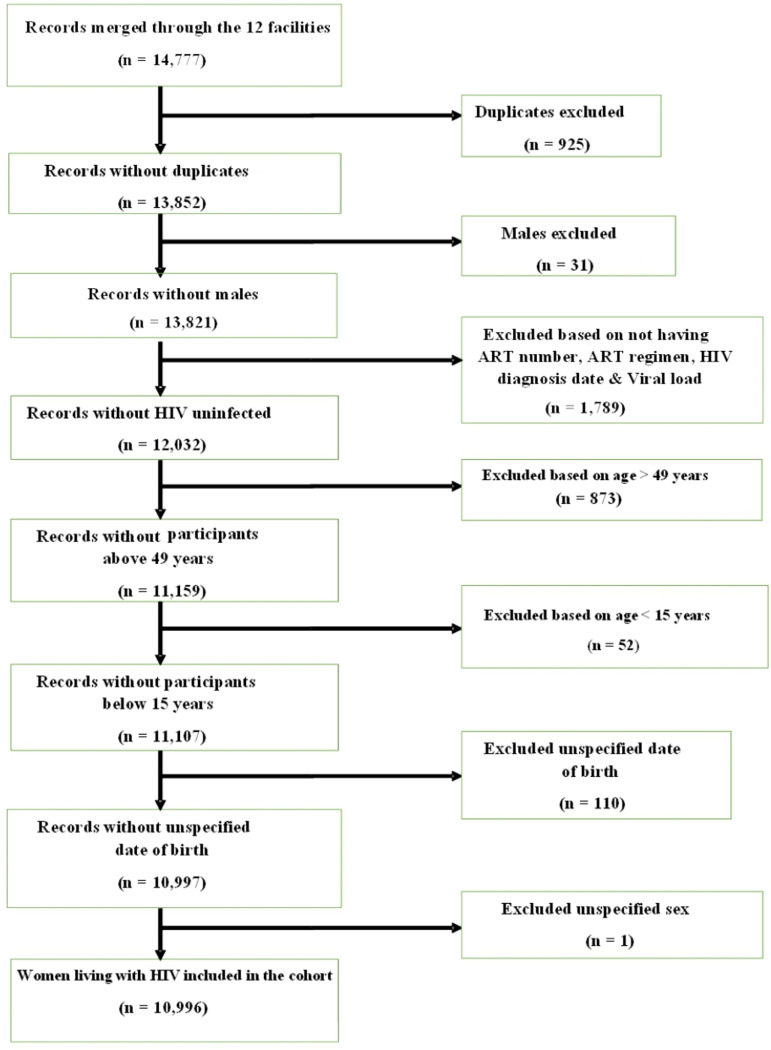
Flow chart for cleaning of extracted data.

A total of 18,210 women living with HIV were recruited in the 12 facilities, representing 51.6% of the expected pool of 35,312 women living with HIV. Those not approached were either lost to follow-up, had transferred out, or were dead before the recruitment. For those approached, some were excluded because they did not meet the eligibility criteria (did not have a functional phone number or did not provide consent). Eighty-one percent (14,777/18,210) of these women had their data extracted from the EMR (Table 1), the rest were either duplicates or HIV-negative women who gave consent. Analysis was restricted to the data of 10,996 women from the 12 facilities after data for 3742 women were excluded due to identified duplicates, no documentation of HIV status, women outside the reproductive age range, and some identified males as shown in Fig 1.

**Table 1:** Number of eligible women living with HIV, consent and extracted data from selected sites in Nigeria.

S/N	Name of Health Facility	Number of eligible women living with HIV	Number that declined consent	Incomplete consent	Number of women living with HIV who gave consent (%) n=18,210	Extracted data (%) n=14,777
1	Annunciation Specialist Hospital, Emene, Enugu State	2,679	0	2	2,276 (12.50)	1,756 (11.88)
2	Mother of Christ Specialist Hospital, Ogui, Enugu	2,583	0	2	951 (5.22)	803 (5.43)
3	Dalhatu Araf Specialist Hospital, Lafia, Nasarawa State	3,455	7	2	2,684 (14.74)	2,333 (15.79)
4	Faith Alive Foundation, Jos, Plateau State	4,329	0	1	2,742 (15.06)	1,919 (12.99)
5	General Hospital, Alimosho, Lagos State	3,154	18	1	950 (5.22)	939 (6.35)
6	State Hospital - Ijebu Ode, Ogun State	2,809	0	1	983 (5.40)	826 (5.59)
7	Calabar General Hospital, Calabar, Cross River State	3,052	0	2	947 (5.20)	733 (4.96)
8	Oron General Hospital, Oron, Akwa Ibom State	2,930	89	1	867 (4.76)	821 (5.56)
9	Dr. Gwamna Awan General Hospital, Kaduna, Kaduna State	3,049	1	2	2,550 (14.00)	23,56 (15.94)
10	General Hospital, Funtua, Katsina State	2,244	0	10	1,635 (8.98)	1,597 (10.81)
11	Gombe State Specialist Hospital, Gombe, Gombe State	3,206	0	10	897 (4.93)	391 (2.65)
12	General Hospital, Billiri, Gombe State	1,822	2	5	728 (3.10)	303 (2.05)
	Total	**35,312**	**117**	**39**	**18,210**	**14,777**

The baseline socio-demographic characteristics of the women living with HIV are shown in Table 2. The mean age was 37.4 ± 7.2 years, with women in age groups 30–39 years and 40–49 years making up 85% of the cohort. Of the participants with reported marital status, education, pregnancy and parity, a greater majority were married (64%), about two thirds had completed at least secondary education (67%), almost all were not pregnant at the time of consent (99%) and approximately one-third (35%) had not experienced childbirth previously. The clinical characteristics of participants (Table 3) shows that mean weight was 66.1 ± 15.6 kg, while mean height was 161.0 ± 8.3 cm. BMI was calculated for the 9718 women with complete values for weight and height. Roughly one in five (19%) of participants were obese with BMI ≥30 kg/m^2^. The median time since HIV diagnosis was 8 years (IQR 3–11 years) while the median length of time on ART was 6 years (IQR 3–10 years). For women who had a record of WHO clinical staging and most current viral load, the majority (80%) were in WHO stage 1 while two thirds (68.0%) had viral load of <20 copies/mm^3^. Almost all women (94%) were on the first-line ART regimen, with none on the third-line regimen. Majority of the women on first line regimen were on Dolutegravir drug combination (TDF/3TC/DTG) while those on second line regimen were mostly on Atazanavir/ritonavir drug combinations (TDF/3TC/ATV/r followed by AZT/3TC/ATV/r).

**Table 2:** Socio-demographic characteristics of women in the NISA-WIC (n=10,996).

Socio-demographic characteristic	Frequency (%)
Age (years) Mean (SD*)	37.4 (7.2)
Age category (years)	
15–19 years	111 (1.0)
20–29 years	1527 (13.9)
30–39 years	4793 (43.6)
40–49 years	4565 (41.5)
Marital status,	
Unmarried	1957 (17.8)
Married	7010 (63.8)
Widowed	953 (8.7)
Separated/Divorced	521 (4.7)
Missing	555 (5.0)
Completed Education,	
No education	797 (7.2)
Primary	1856 (16.9)
Secondary	6389 (58.1)
Tertiary	974 (8.9)
Missing	980 (8.9)
Pregnancy status	
Pregnant	92 (0.8)
Not Pregnant	10889 (99.0)
Missing	15 (0.1)
Parity	
0	3881 (35.3)
1-3	3867 (35.2)
4-6	1913 (17.4)
>7	168 (1.5)
Missing	1167 (10.6)

*SD – Standard deviation.

**Table 3:** Clinical characteristics of women in the NISA-WIC.

Clinical Characteristic	Frequency (%)
Weight Kg, mean (SD*) *n* = 10953	66.1 (15.6)
Height m, mean (SD) *n* = 9770	161.0 (8.3)
BMI (Kg/m^2^) n = 9718	
<18.5	864 (8.9)
18.5-24.9	4243 (43.7)
25-29.9	2782 (22.6)
30-34.9	1205 (12.4)
35-39.9	398 (4.1)
≥40	226 (2.3)
Time since HIV diagnosis (years), median (IQR) n= 10111	8 (3 - 11)
Time on ART (years), median (IQR^#^) n= 10,969	6 (3 - 10)
*Current WHO stage,*	
Stage 1	8785 (80.0)
Stage 2	1039 (9.4)
Stage 3	334 (3.0)
Stage 4	142 (1.3)
Missing	696 (6.3)
*Current viral load copies/ml,*	
<20 copies	7525 (68.4)
20–1000 copies	2794 (25.4)
>1000 copies	436 (4.0)
Missing data	241 (2.2)
*ART regimen*	
First-line	10326 (93.9)
Second-line	660 (6.0)
Missing	10 (0.1)

*SD – Standard deviation; IQR^#^ – Interquartile range.

## Discussion

The NISA WIC’s profile differed from other cohorts from similar settings due to differences in the variables reported. In an earlier cohort in Nigeria, MoMent Nigeria, only age, education, and marital status were comparable and there were marked differences [11]. This could be because the MoMent Nigeria study was among only pregnant women living with HIV. Other studies such as the Nigeria HIV/AIDS Indicator and Impact Survey (NAIIS) reported data for both women living with HIV and those who were not while our study focused on only women living with HIV. The NAIIS data for women living with HIV also had a different focus compared to this study. Their data on women living with HIV such as those on ART, focused on the number who received ARVs reported by residence, marital status, education, and type of union. Ours reported the line of regimen for those on ARVs [31]. Other recent cohorts of people living with HIV were not comparable as they were not focused on women of reproductive age [32–34].

The contributions of cohort studies to public health cannot be overstated. Cohort studies have been instrumental in identifying the causes and risk factors for various diseases. Establishing causal relationships and understanding disease mechanisms can be achieved through cohort studies when large groups of people are observed over time in relation to different exposures. Cohort studies provide valuable data on the effectiveness of preventive interventions. For example, they can assess the long-term impact of vaccines, public health campaigns, or lifestyle changes on disease incidence [35]. In addition, findings from cohort studies often contribute to the development of clinical guidelines and health policies, as policymakers and healthcare providers use the evidence generated by such studies to make informed decisions that improve population health outcomes [35]. It is pertinent to note that while large population based cohorts exist in many western countries [36,37], these studies are sparse in sub-Saharan Africa (SSA) [38,39]. Increasing the number of cohorts in SSA that include women and children provides an opportunity to generate unique data for collaborative discovery science and to understand factors associated with maternal, fetal, and neonatal morbidity and mortality [40].

The NISA-WIC is a unique prospective cohort in Nigeria. This cohort will provide researchers a platform to propose and answer several research questions about the health of women and infants providing policymakers with information on maternal and child health in Nigeria. For instance, a cohort of women living with HIV holds significant value in several aspects of research, public health, and clinical practice. Firstly, researchers can gain more insights into the natural history of HIV and how it progresses in women specifically. This includes understanding the impact of HIV on all aspects of women’s health, the development of co-morbid conditions such as cardiovascular disease, cancers, and opportunistic infections, and how these conditions interact with HIV [41]. Secondly, this cohort can provide valuable data on the long-term effectiveness and side effects of antiretroviral therapies (ART) in women, while keeping track of adherence to medication [42]. Thirdly, studying a cohort of women living with HIV, particularly those who are pregnant or of childbearing age, is essential for a better understanding of the transmission of HIV from mother to child and for developing strategies to prevent it. This includes assessing the effectiveness of ART in reducing vertical transmission of HIV, understanding the outcomes of uninfected but HIV-exposed children, and improving maternal health care to enhance both maternal and child health outcomes [43].

Although other PMTCT cohorts exist in Nigeria [11,44], the NISA-WIC will be the first, large, nationally representative cohort consisting of women in the reproductive age group. This cohort will create a robust database to enable collation of data that provide a representative view across the six geopolitical regions of the country, thereby yielding results that are more generalizable to the larger population. This cohort will also serve as a foundation to enable Nigeria to further understand her HIV epidemic, drive policy formulation, and aid decision making on MTCT, thus drawing the country closer to achieving the UNAIDS 2030 Fast-Track targets [45].

The strengths of this cohort study lie in its representativeness and the potential size of the cohort. The joint efforts of experts from implementing partners, HIV treatment facilities, academia, and government institutions, is another important strength of this cohort that will support its sustainability. Additionally, since the cohort is funded domestically, it is less likely to be disrupted as reported with another cohort in SSA [46]. Furthermore, the NISA-WIC is set up to strengthen the linkage between medical researchers, community clinicians and the communities they serve, ensuring that stakeholder engagement is optimal, thus contributing to the successful integration of evidence-based practices within healthcare organizations [47].

This cohort has some limitations. As with other observational cohorts, poor quality data, absence of data on potential confounders and participant loss-to-follow-up [48,49] may pose significant challenges. However, it also provides an opportunity to improve data quality at the selected sites through frequent training and supportive supervision of staff which can translate to data quality benefits in future cohort studies. To ensure sustainability of the cohort, the facilities will be supported through the implementing partners to provide close monitoring to reduce cases of loss-to-follow-up (LTFU) following the National Guideline for HIV Prevention, treatment, and Care [50]. The chronicity of HIV with the need for clinic and lab visits in addition to tracking of defaulters by the health providers will help in maintaining this cohort through the long observation period. Issues with resource constraints will be mitigated by leveraging the NISA partnerships as well as applying for research grants. Notwithstanding these potential limitations, the NISA-WIC offers a unique opportunity to generate reliable evidence for maternal and child health issues and other chronic diseases in Nigeria and Sub-Saharan Africa.

## Conclusion

The NISA-WIC in Nigeria represents a significant advancement in public health research, particularly concerning women’s and infants’ health. Cohort studies, such as that conducted with the NISA-WIC, are invaluable for identifying causes and risk factors for diseases by observing large groups over time. Findings from these cohort studies can influence the development of clinical guidelines and health policies, thus improving population health outcomes. However, there is a notable scarcity of such studies in sub-Saharan Africa, highlighting the importance of the NISA-WIC. Ultimately, the NISA-WIC will support policy formulation and decision-making, aiding Nigeria in its efforts to achieve UNAIDS 2030 Fast-Track targets.
